# Supplementary material for: Inclusion of Soluble Fiber in the Gestation Diet Changes the Gut Microbiota, Affects Plasma Propionate and Odd-Chain Fatty Acids Levels, and Improves Insulin Sensitivity in Sows
Source: Int J Mol Sci. 2020 Jan 18;21(2):635. doi: 10.3390/ijms21020635 (PMC7013540; doi:10.3390/ijms21020635)
Supplement: Supplementary file 1 [file ijms-21-00635-s001.zip › ijms-684526-SI.doc]

**Supplementary Table S1.** Ingredients and nutrient composition of experimental gestation and lactation diets (as-fed basis)

| **Items** | **Gestation diet** | | **Lactation diet** |
| --- | --- | --- | --- |
|  | **Control** | **SF^1^** |  |
| Ingredient, % |  |  |  |
| Corn | 54.80 | 54.80 | 54.40 |
| Soybean meal, 43%CP | 11.50 | 11.50 | 26.00 |
| Wheat bran | 0.00 | 0.00 | 11.00 |
| Soybean hull | 14.00 | 14.00 | 0.00 |
| Rice bran meal | 16.00 | 14.00 | 0.00 |
| Calcium carbonate | 1.20 | 1.20 | 1.51 |
| Dicalcium phosphate | 1.00 | 1.00 | 1.23 |
| Salt | 0.40 | 0.40 | 0.26 |
| Mildewcide^2^ | 0.10 | 0.10 | 0.10 |
| Choline chloride | 0.00 | 0.00 | 1.00 |
| Fiber source | 0.00 | 2.00 | 0.00 |
| Premix^3^ | 1.00 | 1.00 | 1.50 |
| Nutrient composition |  |  |  |
| Net energy, MJ/kg^4^ | 9.53 | 9.53 | 10.36 |
| Crude protein, % | 13.96 | 13.81 | 18.92 |
| ISF, %^5^ | 26.95 | 26.78 | 14.45 |
| SF, %^6^ | 2.85 | 4.04 | 1.76 |
| NDF, %^7^ | 20.29 | 20.21 | 11.18 |
| ADF, %^8^ | 5.47 | 5.42 | 4.26 |
| Lys, %^4^ | 0.59 | 0.59 | 1.03 |
| Ca, %^4^ | 0.78 | 0.75 | 1.06 |
| Available P, %^4^ | 0.40 | 0.40 | 0.45 |

^1^SF = 2% fiber of pregelatinized waxy maize starch (85.7%) plus guar gum (14.3%).

^2^Mildewcide: ammonium propionate.

^3^Provided per kg of diet: Cu, 30 mg; Fe, 160 mg; Zn, 160 mg; Mn, 55 mg; I, 0.5 mg; Se, 0.5 mg; Co, 0.8 mg; Cr, 0.2 mg; Vitamin A, 14000 IU; Vitamin D_3_, 2900 IU; Vitamin E, 120 mg; Vitamin K_3_, 6 mg; Vitamin B_1_, 2.4 mg; Vitamin B_2_, 8.5 mg; Vitamin B_6_, 4.5 mg; Vitamin B_12_, 0.03 mg; Vitamin H, 0.55 mg; Pantothenic acid, 30 mg; Folic acid, 5 mg; Nicotinamide, 50 mg.

^4^Calculated chemical concentrations using values for feed ingredients from the NRC (2012).

^5^insoluble fiber.

^6^solubel fiber.

^7^neutral detergent fiber.

^8^acid detergent fiber.
